# Detection of *Staphylococcus aureus* enterotoxigenic strains in bovine raw milk by reversed passive latex agglutination and multiplex polymerase chain reaction

**DOI:** 10.14202/vetworld.2017.843-847

**Published:** 2017-08-01

**Authors:** Asmaa Samy Mansour, Gad El-Said Wagih, Sabry D. Morgan, Mahmoud Elhariri, Mona A. El-Shabrawy, Azza S. M. Abuelnaga, E. A. Elgabry

**Affiliations:** 1 Department of Microbiology and Immunology, National Research Centre, Dokki, Giza, Egypt; 2 Department of Microbiology, Faculty of Veterinary Medicine, Cairo University, Giza, Egypt; 3 Department of Milk Hygiene and Control, Faculty of Veterinary Medicine, Cairo University, Giza, Egypt

**Keywords:** enterotoxin genes, multiplex polymerase chain reaction, reversed passive latex agglutination, raw milk, *S. aureus*

## Abstract

**Aim:**

This review gives an outline of the assessment of enterotoxigenic *Staphylococcus aureus* tainting levels in raw milk from different sources in Egypt and characterization of enterotoxigenic strains utilizing a technique in light of PCR to identify genes coding for the production of staphylococcal enterotoxin (SE). The obtained data were compared with results from the application of the reversed passive latex.

**Materials and Methods:**

Multiplex PCR and reversed passive latex agglutination (RPLA) were used. A total of 141 samples of raw milk (cow’s milk=33, buffalo’s milk=58, and bulk tank milk=50) were investigated for *S. aureus* contamination and tested for enterotoxin genes presence and toxin production.

**Results:**

*S. aureus* was detected in 23 (16.3%) samples phenotypically and genotypically by amplification of *nuc* gene. The *S. aureus* isolates were investigated for SEs genes (*sea* to *see*) by multiplex PCR and the toxin production by these isolates was screened by RPLA. SEs genes were detected in six isolates (26.1%) molecularly; *see* was the most observed gene where detected in all isolates, two isolates harbored *seb*, and two isolates harbored *sec*. According to RPLA, three isolates produced SEB and SEC.

**Conclusion:**

The study revealed the widespread of *S. aureus* strains caring genes coding for toxins. The real significance of the presence of these strains or its toxins in raw milk and their possible impact a potential hazard for staphylococcal food poisoning by raw milk consumption. Therefore, detection of enterotoxigenic *S. aureus* strains in raw milk is necessary for consumer safety.

## Introduction

The tracking of sentinel health events to detect and manage disease risks facing a human population is an important mission. Yet the full potential of linking animal and human health information to provide warning of such “shared risks” from environmental hazards has not been realized [1]. Animal or food of animal origin acting as a potential human health hazard [2-5].

Milk is an important food because it contains numerous important nutrients including proteins, vitamins, and minerals. On the other hand, *Staphylococcus aureus* is the most common microorganism incriminated in staphylococcal food poisoning because it is considered a principal contaminant of raw milk [6,7].

Staphylococcal foodborne poisoning is caused by the ingestion of food containing staphylococcal enterotoxins (SEs). Symptoms include nausea, vomiting, abdominal cramps, and diarrhea. The effect of symptoms is rarely severe, leading to high levels of under-reporting. The classical antigenic-based classification of SEs includes five classical types: SEA, SEB, SEC, SED, and SEE. In latest years, new types of SEs (SEG, SEH, SEI, SEJ, SEK, SEL, SEM, SEN, SEO, SEP, SEQ, SER, and SEU) have been reported by Riva *et al*. [8]. Other enterotoxins have been discovered as SET [9], SElV [10], and SElX [11]. These new toxins have been identified based on their sequence similarity with classical SEs. SEG, SEH, and SEI were tested, and their emetic properties were confirmed [12].

SEs are small proteins (MW 26.900 - 29.600 KD) [13]. They resist the majority of proteolytic enzymes and thus remain their action in the gastrointestinal tract. SEs are highly heat resistant [14]. They retain their biological activity even after pasteurization; staphylococcal enterotoxin A (SEA), for example, keeps some activity after 28 min at 121°C [15]. The quantity of SEs is needed for appearance of food poisoning symptoms is very small (20 ng to 1 µg) which is produced by about 10^5^ CFU of *S. aureus*/g of food [16].

Considering these facts, the present work studied: (i) The occurrence of genes coding the SEs (SEA, SEB, SEC, SED, and SEE) using multiplex polymerase chain reaction (PCR) and (ii) production of SEs using reversed passive latex agglutination (RPLA) technique.

## Materials and Methods

### Ethical approval

All the samples were collected and complies with relevant legislation. It follows the international guiding principles for biomedical research involving animals.

### Sampling

A total of 141 samples of raw milk (cow milk, n=33; buffalo milk, n=58; bulk tank milk, n=50) were collected randomly from farms (n=4) and markets (n=50) in different governorates in Egypt (Cairo, Giza, Kafr El-Sheikh). All the samples were taken to the laboratory under refrigerate conditions.

### Isolation and identification of *S. aureus*

Each milk sample was cultured directly on mannitol salt agar (Lab M) and incubated at 37°C for 24 h [17]. One colony from each sample was tested for catalase, coagulase [18], and thermonuclease production [19]. The positive species were submitted to the Voges-Proskauer test (MR-VP broth, Oxoid, England) [20] to discriminate *S. aureus* (positive) from *Staphylococcus intermedius* (negative).

### DNA extraction

Enterotoxigenic *S. aureus* strains ATCC 13565 (SEA), ATCC 14458 (SEB), ATCC 19095 (SEC), FRI 361 (SED), and ATCC 27664 (SEE) were used as positive controls [15].

DNA was extracted using a genomic DNA purification kit (Qiagen, Germany) according to the manufacturer’s recommendations. The primers used for the detection of SE genes are listed in Table 1 [21-24].

**Table-1:** Primers used for the detection of *Staphylococcus aureus* (SE) genes.

Gene	Primer	Sequence	Base pair	References
*nuc*	*nuc*-F	5′GCGATTGATGGTGATACGGTT 3′	279	[21]
*sea*	*sea*-F	5′ GAAAAAAGTCTGAATTGCAGGGAACA3′	561	[22]
*seb*	*seb*-F	5′ TCG CAT CAA ACT GAC AAA CG 3′	478	[23]
*sec*	*sec*-F	5′ GAC ATA AAA GCT AGG AAT TT 3′	257	[23]
*sed*	*sed*-F	5′ CTA GTT TGG TAA TAT CTC CT 3′	317	[23]
*see*	*see*-F	5′ TAGATAAAGTTAAAACAAGC 3′	170	[24]

SE=Staphylococcal enterotoxin

### Molecular identification of *S. aureus* by amplification of *nuc* gene

The reaction mixture contained 12.5 µl Master mix, 4 µl target DNA, 0.5 µl of primers, 2 µl MgCl_2_, and the final volume was adjusted to 25 µl by adding sterile distilled water. Amplification was carried out in a thermocycler (Esco-Swift MiniPro) with the following thermal cycling profile: Initial denaturation at 94°C for 10 min was followed by 35 cycles of amplification (denaturation at 94°C for 1 min, annealing at 52°C for 1 min, and extension at 72°C for 1 min), ending with a final extension at 72°C for 10 min.

### PCR testing for genes encoding SEs (SEA to SEE)

The PCR reaction mixture performed as follow 12.5 µl PCR DreamTaq Green PCR Master Mix (2X) (Thermo), 10 pmol of each primer. The final volume was adjusted to 25 µl by adding sterile ultrapure water. DNA amplification was performed in a (Esco-Swift MiniPro) thermal cycler using the following conditions: Initial denaturation for 5 min at 94°C followed by 30 cycles of denaturation (94°C for 2 min), annealing, and extension (72°C for 1 min). Different annealing temperatures were tested as shown in Table 1. A final extension step (72°C for 5 min). The amplified PCR products were separated by electrophoresis. 15 µl of each PCR product was mixed with 6× loading buffer then the PCR products were run in parallel with a 100 bp ladder molecular weight marker on a 1.5% agarose gel (Sigma-Aldrich) in tris acetate EDTA (TAE) (Sigma-Aldrich) stained with 10 μl ethidium bromide. The PCR products were run for 30 min at about 100 V.

### SE production test by SET-RPLA

*S. aureus* isolates were tested for enterotoxin production (SEA to SED) by SET-RPLA assay (Oxoid). The isolates were cultured on brain heart infusion agar (Oxoid, England) slant and incubated for 18-24 h at 37°C then harvested with 2 ml sterile saline and 8 ml of sterile phosphate buffer saline [25]. Testing with SET-RPLA was thereafter performed according to the manufacturer’s instructions.

## Results

**Figure-1:**
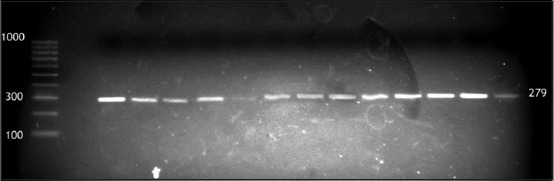
Electropherotic profile of *Staphylococcus aureus* isolates positive *nuc* gene 279 bp, DNA marker 100 bp (Jena Bioscience).

**Figure-2:**
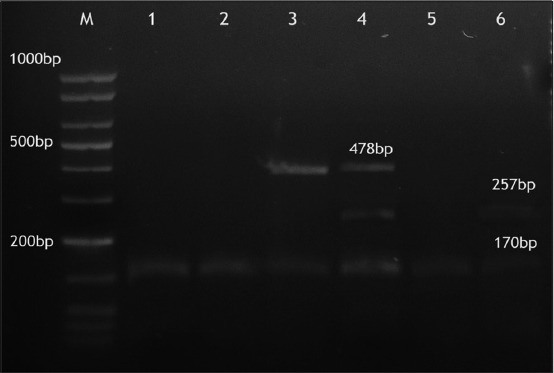
Enterotoxin genotyping pattern of examined six *Staphylococcus aureus* strains, DNAmarker low range 50 bp (Jena Bioscience).

Out of 141 raw milk samples, 23 isolates of *S. aureus* were detected (16.3%); 3 out of 33 cow milk, 3 out of 58 buffalo milk, and 17 out of 50 bulk tank milk. *S. aureus* isolates were confirmed using PCR by amplification of thermonuclease (*nuc)* gene (Figure-1). Multiplex PCR was used for the detection of genes encoding SEA, SEB, SEC, SED, and SEE for the 23 strains of *S. aureus* tested, 6 (26.1%) were positive for one or more SE genes as illustrated in (Figure-2).

Three strains expressed only one enterotoxin gene (*see*); 2 strains carried genes coding for two enterotoxins patterns (*see*, *seb*) and (*see*, *sec*) and only one strain carrying three genes (*seb*, *sec*, *see*). The two genes encoding *sea* and *sed* were not observed (Table-2). All over four different enterotoxins genotyping pattern were observed.

**Table-2:** Enterotoxins genotypic profile of *S. aureus* isolates in relation to *in vitro* production of classical enterotoxins (SEA, SEB, SEC and SED), as detected by the SETRPLA.

Isolate No.	*sea*	*seb*	*sec*	*sed*	*see*	Enterotoxin genotyping pattern	SET-RPLA
Isolate 1					+	*see*	-
Isolate 2					+	*see*	-
Isolate 3		+			+	*seb, see*	SEB
Isolate 4		+	+		+	*seb, sec, see*	SEB, SEC
Isolate 5					+	*see*	-
Isolate 6			+		+	*sec, see*	SEC

SE=Staphylococcal enterotoxin, RPLA=Reversed passive latex agglutination, *S. aureus=Staphylococcus aureus*

The most frequent gene was *see* and it was found in all isolates (100%), followed by *seb* and *sec* in two isolates each and none of the isolates harbored *sea* or *sed* as shown in Table 2. The classic enterotoxin production was screened by RPLA assay and it was found that 3 out of 23 isolates produced enterotoxins: One produced SEB, one produced SEC, one produced SEB in combination of SEC, and none of the isolates produced SEA or SED as in Table 2.

## Discussion

Many scientific literature focusing on animals and humans share risk of exposure to toxins or infectious agents. These events highlight the value of animals as potential hazard for human health, which evoke the need to systematically compare animal and human health surveillance data [1-6].

*S. aureus* is one of the predominant microorganisms present in raw milk. Milk is a good medium for the multiplication of this bacterium especially with reduced hygienic measures and decrease of the cooling services [26].

In this study, raw milk samples were screened for the presence of *S. aureus* carrying enterotoxins genes. Out of 141 raw milk samples were contaminated with 23 isolates of *S. aureus* (16.3%), and this percent of contamination by *S. aureus* were encountered by many studies [27-31]. On the other hand, lower recovery of *S. aureus* was reported by Rahimi and Alian [32], Fagundes *et al*. [33] and Mørk *et al*. [34]. On the other hand, higher contamination rates have also been found [35-39]. The contamination of milk can be internally through the secretion of milk from the infected animal or externally through the infected persons (approximately, 50% of the human population carries *S. aureus* as commensals) or through the environment (soil, water, dust, and air) [40]. Milk and milk products are widely consumed since ancient times and its market demand is continuous worldwide [41].

*S. aureus* microorganisms have the ability to produce the enterotoxins which make a risk factor on public health [42].

Different techniques are used for detection of *S. aureus* strains producing enterotoxins phenotypically and genotypically. The phenotypic characterization is not specific, because SEs types are nearly similar in genomic structure [43]. Commercial RPLA kits were not able to detect all the different types of enterotoxins and are specific to (SEA, SEB, SEC, and SED). Molecular detection by multiplex PCR was used for detection of genes encoding enterotoxins of *S. aureus*, but the enterotoxin gene presence does not consider a sign for its production. Therefore, the toxin production should also be tested by RPLA technique [44].

With regard to the genes encoding enterotoxins, 26.1% (6 isolates) of all detected isolates displayed the presence of SEs genes with *see* being the most frequent gene (Table 2). The relatively high percentage of enterotoxigenic *S. aureus* strains from milk samples found in this study is confirmed by previous findings [29,45].

However, these results were not in agreement with some other authors. Higher percentages were reported in other studies [8,39] and lower rates were also found [46,47].

The classical enterotoxin, SEE, has been infrequently reported in foods and food-producing animals. It was recorded six staphylococcal food poisoning outbreaks, which occurred in France at the end of 2009, were caused by SEE present in soft cheese made from unpasteurized milk. This enterotoxin has also been associated with outbreaks in the USA and UK [48-52].

Enterotoxin production is due to the presence of the corresponding genes. Three out of 23 *S. aureus* isolates produced classic enterotoxins (SEB, SEC). 17 strains, negative by PCR were also negative in the SET-RPLA assay. The results of PCR technique agreed with the results of RPLA technique in concern of the classic enterotoxins.

This study obviously indicated that milk was contaminated with *S. aureus*, posing a high risk of food poisoning. More detailed studies are needed on the occurrence of newly discovered SE gene because of contamination of milk with new enterotoxigenic strains of this bacterium are increasingly being reported in many other parts of the world [38].

## Conclusion

This study detected the presence of enterotoxin genes, and toxin production by *S. aureus* isolates from raw milk. This considered a potential risk for food poisoning by raw milk consumption. Therefore, the rapid and efficient detection of enterotoxigenic *S. aureus* strains in raw milk is necessary for consumer safety. Multiplex PCR techniques, allowing rapid and simultaneous detection of enterotoxigenic strains, gave good results in agreement with RPLA.

## Authors’ Contributions

ASM collected the samples, carried out the laboratory work. ME achieved the molecular work of the study. EAE drafted the manuscript, supervised the research work and revised the manuscript. GEW, SDM and MAE provided guidance for the research work. ASMA and EAE revised the manuscript. All authors read and approved the final manuscript.
